# An indium-111-labelled membrane-targeted peptide for cell tracking with radionuclide imaging

**DOI:** 10.1039/d2cb00164k

**Published:** 2022-10-19

**Authors:** Johanna Pruller, Truc Thuy Pham, Julia E. Blower, Putthiporn Charoenphun, Alessia Volpe, Kavitha Sunassee, Gregory E. D. Mullen, Philip J. Blower, Richard A. G. Smith, Michelle T. Ma

**Affiliations:** a Randall Division of Cell and Molecular Biophysics, King's College London UK; b School of Biomedical Engineering and Imaging Sciences, King's College London, St Thomas’ Hospital London UK michelle.ma@kcl.ac.uk; c Department of Diagnostic and Therapeutic Radiology, Faculty of Medicine, Ramathibodi Hospital, Mahidol University Bangkok Thailand; d MRC Centre for Transplantation, King's College London, Guy's Hospital London UK

## Abstract

Cell labelling agents that enable longitudinal *in vivo* tracking of administered cells will support the clinical development of cell-based therapies. Radionuclide imaging with gamma and positron-emitting radioisotopes can provide quantitative and longitudinal mapping of cells *in vivo*. To make this widely accessible and adaptable to a range of cell types, new, versatile and simple methods for directly radiolabelling cells are required. We have developed [^111^In]In-DTPA-CTP, the first example of a radiolabelled peptide that binds to the extracellular membrane of cells, for tracking cell distribution *in vivo* using Single Photon Emission Computed Tomography (SPECT). [^111^In]In-DTPA-CTP consists of (i) myristoyl groups for insertion into the phospholipid bilayer, (ii) positively charged lysine residues for electrostatic association with negatively charged phospholipid groups at the cell surface and (iii) a diethylenetriamine pentaacetate derivative that coordinates the γ-emitting radiometal, [^111^In]In^3+^. [^111^In]In-DTPA-CTP binds to 5T33 murine myeloma cells, enabling qualitative SPECT tracking of myeloma cells’ accumulation in lungs immediately after intravenous administration. This is the first report of a radiolabelled cell-membrane binding peptide for use in cell tracking.

## Abstract 2

An indium-111-labelled peptide binds to myeloma cells, enabling SPECT imaging of myeloma cell accumulation in lungs after intravenous administration. This is the first report of a radiolabelled cell-membrane binding peptide for cell tracking.

## Graphical abstract

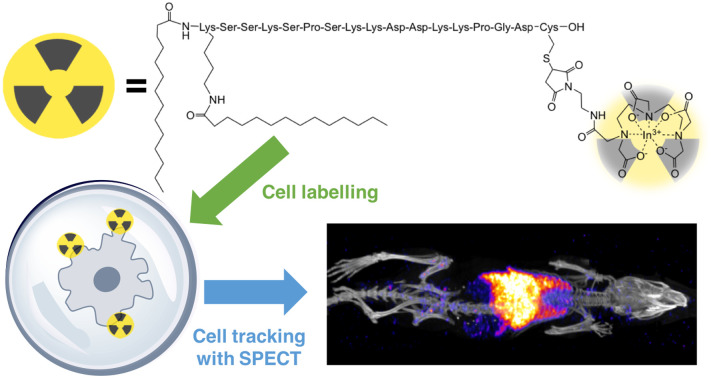

## Background

Cell-based therapies are potentially transformative in oncological,^1^ cardiac,^2^ neurological,^3^ transplant^4^ and regenerative therapies.^5^ Chimeric Antigen Receptor (CAR) T-cells for treatment of leukaemia and lymphoma have recently entered the clinic, with durable responses in some patients.^1,6^ The ability to track the migration and distribution of therapeutic cells in a living organism will (i) aid development of cell-based therapeutics, (ii) allow clinical assessment of migration and safety of small populations of cells in patients before administering larger doses, and (iii) help predict efficacy of cellular therapies and understand outcomes. Whole body *in vivo*, non-invasive cell tracking methods can give real-time information about the distribution of administered cells.^7^ Additionally, *in vivo* imaging of tumor cells labelled with contrast agents gives detailed information about their metastatic potential in animal models.^8^ In direct cell labelling approaches, cells take up contrast agent prior to being administered to subjects or patients. Magnetic resonance imaging (MRI) (with nanoparticles^9^) and optical imaging (with fluorophores^10^) have enabled whole body imaging of cell distribution. However, MRI requires large amounts of contrast agent, and optical methods are hampered by low tissue penetration of light for excitation and emission of fluorophores. Neither method is quantitative. Radionuclide imaging methods (Single Photon Emission Computed Tomography (SPECT) and Positron Emission Tomography (PET)) are both quantitative and more sensitive than magnetic resonance and optical imaging.^11^

There are several strategies for direct radiolabelling of cells for PET and SPECT imaging. They include radiolabelled receptor-targeted compounds that are taken up by cell surface receptors (*e.g.* [^18^F]FDG targeting GLUT,^12^ radiolabelled antibodies^13^), radiolabelled compounds with a pendant reactive group for covalent linkage to cell membrane proteins/glycoproteins (*e.g. via* endogenous primary amines^14^ or thiols^15^), radiolabelled nanoparticles that internalise in cells,^16^ and lipophilic, neutral radiometallic complexes (ionophores) that diffuse across cell membranes and dissociate, releasing their radioactive cargo inside the cell.^17–20^ Ionophores including [^111^In]In-(oxine)_3_^19^ and [^99m^Tc]Tc-HMPAO^20,21^ have been in clinical use since the 1980s to radiolabel leukocytes, for administration to patients to image inflammation and infection with γ-scintigraphy or SPECT. Lipophilic zirconium-89- and copper-64-labelled ionophores for PET cell tracking have also been developed.^17,18,22,23^ Lastly, in recent years, elegant methods for indirect radiolabelling of cells have been developed: in this approach, cells are genetically modified with a reporter gene that leads to expression of a cell-surface receptor.^24–28^ The cells can then be administered to a subject or patient. A complementary radiotracer with high specificity and affinity for the receptor is subsequently used to track this introduced cell population. There are many advantages to this approach. First, the cell population can be tracked over an indefinite period of time, simply by administration of the radiotracer followed by imaging. In direct cell labelling, longitudinal cell tracking is limited by the half-life of the selected radioisotope. Second, provided that reporter gene expression is stable, this approach allows imaging and quantification of expansion or proliferation of the cell population *in vivo*. In instances where populations of cells require genetic manipulation to elicit a therapeutic response (*e.g.* CAR T-cells^24,26–28^), introduction of a reporter gene to enable radionuclide imaging is viable. However, in other instances (*e.g.* leukocyte imaging), the practical complexity of genetic manipulation of cells (including GMP manufacturing and safety management) is undesirable.

Herein we describe a new approach for direct radiolabelling of cells,^25^ utilising a synthetic “cytotopic” peptide (CTP) that Smith *et al*. have pioneered for attachment of functional cargo to the surface of cell membranes.^29–36^ CTP consists of two long-chain C_13_ myristoyl groups for insertion into cellular lipid bilayers, and multiple lysine residues for favourable electrostatic interactions with negatively charged phospholipid groups of the cell surface membrane.^31,35,37^ We have prepared a radiolabelled chelator bioconjugate of CTP: to the best of our knowledge, this is the first report of a radiolabelled peptidic bioconjugate designed to associate with cell membranes, for cell tracking with radionuclide imaging. Such a construct could have potential advantages over existing cell radiolabelling platforms: (i) it localises the radionuclide to the cell membrane, increasing the distance between the radionuclide and the cell nucleus and thus potentially decreasing toxicity or impaired cellular function associated with intracellular emission of secondary electrons (*e.g.* Auger electrons);^38,39^ (ii) in contrast to methods that covalently append radiolabelled motifs to cell surface proteins,^14,15^ our approach does not involve chemical modification of cell surface proteins, which could be detrimental to cell function and migration; (iii) it is a versatile approach that can be used for radiolabelling many cell types unlike receptor-targeted approaches^13^ that are restricted to only some cell types and cannot be applied when the target receptor is not expressed; and (iv) radiosynthesis does not require a large excess of chelator (as is required for preparations of [^111^In]In-(oxine)_3_, in which traces of oxine can be toxic to cells).

**Fig. 1:**
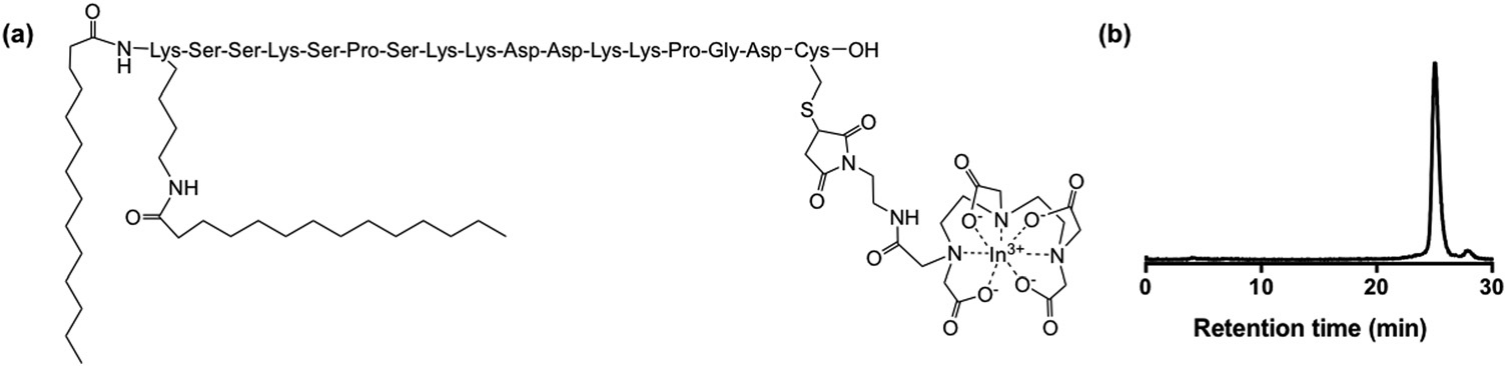
(a) [^111^In]In-DTPA-CTP (b) reverse phase C_8_ HPLC radiochromatogram of [^111^In]In-DTPA-CTP. Radiochemical yield measured >96% at a specific activity of 16 MBq μg^−1^.

The cell membrane binding properties of this class of cytotopic peptide have been well characterised: both hydrophobic and electrostatic interactions contribute to stable cell membrane binding.^35,37^ Cytotopic bioconjugates of the CTP type (Fig. 1(a)) have been used *in vitro* and *in vivo* to anchor proteins that prevent or modulate complement activation,^31,32,36^ thrombosis^30,33^ and hormone signalling^29^ to the cell membrane. They have low systemic toxicity. A CTP–protein bioconjugate of a complement inhibitor is in clinical trials, where it is administered to kidneys prior to transplantation, to decrease transplant rejection and perfusion-related complications.^32^ In an *ex vivo* kidney model, a fluorescent CTP bioconjugate has demonstrated uptake and stable adherence to kidney vasculature in the presence of normal blood flow.^30^ CTP is thus a promising agent for delivery of radioactive cargo to the surface of cells for *in vivo* cell tracking.

## Results

### Preparation of [^111^In]In-DTPA-CTP

We elected to radiolabel CTP with the γ-emitting isotope, indium-111, as this allows direct preclinical comparison with the clinically used cell tracking agent, [^111^In]In-(oxine)_3_. To this end, precursor peptide, with the sequence K(α,ε-bis-myristoyl)-SSKSPSKKDDKKPGD-C(*S*-(2-pyridyldithio))-OH, was reduced with tris(2-carboxyethyl)phosphine to cleave the peptide disulfide bond. The reduced peptide was then conjugated at the thiolate of its C-terminal cysteine to a derivative of the diethylenetriaminepentaacetate (DTPA) chelator that contains a pendant maleimide. DTPA was chosen because it is known to complex radiopharmaceutical concentrations of [^111^In]In^3+^ rapidly and stably at room temperature,^40,41^ for example in [^111^In]In-DTPA-antibody derivatives,^42,43^ and clinically used [^111^In]In-DTPA-octreotide.^44^ DTPA-CTP bioconjugate was purified using a C18 cartridge. Reaction of the pure DTPA-CTP with solutions of [^111^In]In^3+^ (in aqueous 0.2 M ammonium acetate, pH 6) furnished [^111^In]In-DTPA-CTP (Fig. 1(a)) in radiochemical yields of >96% at specific activities of 16 MBq μg^−1^ without need for post-labelling purification (Fig. 1(b)).

### *In vitro* cell binding, viability and retention

The *in vivo* migration of 5T33 murine myeloma cells in immunocompromised mouse models is well characterised,^18,45^ and so these cells were selected for radiolabelling and cell tracking studies with [^111^In]In-DTPA-CTP.

**Fig. 2:**
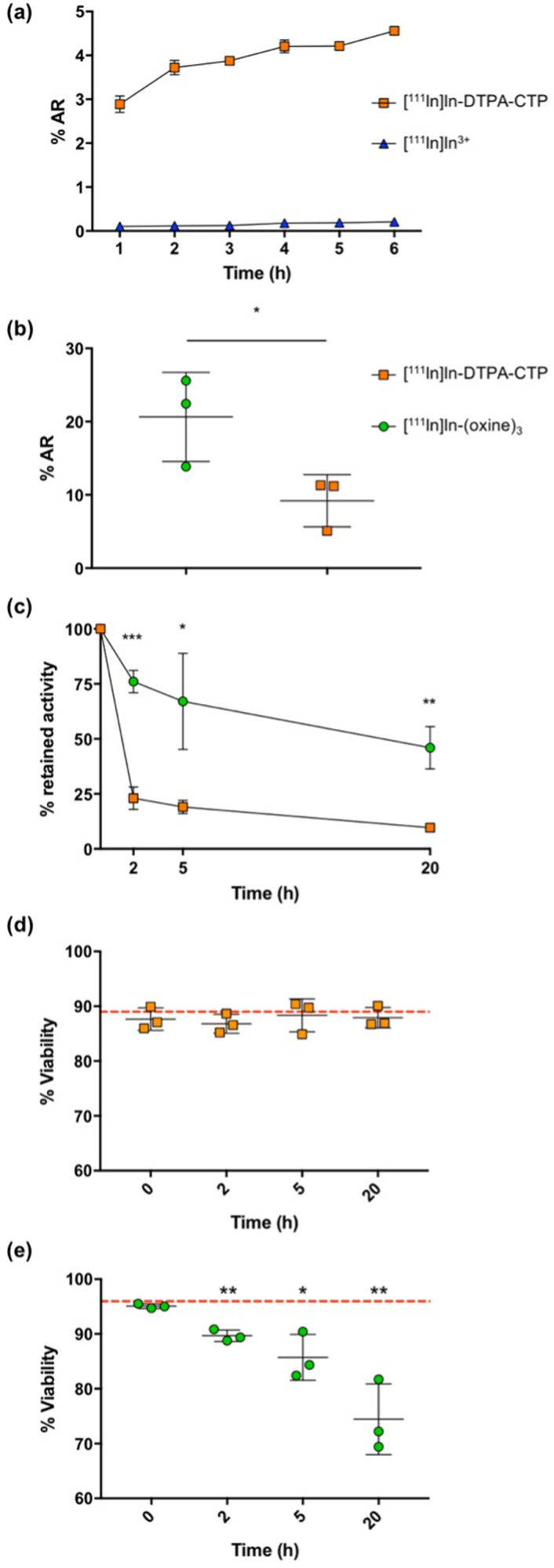
(a) Uptake (percentage added radioactivity, %AR) of [^111^In]In-DTPA-CTP and [^111^In]In^3+^ (each 100 kBq) in 5T33 murine myeloma cells (2 × 10^6^) over 6 h. (b) 5T33 murine myeloma cell uptake of [^111^In]In-DTPA-CTP and [^111^In]In-(oxine)_3_ (10^5^ cells, 2 MBq of added radioactivity). (c) Indium-111 retained by cells, 2, 5 and 20 h after radiolabelling with [^111^In]In-DTPA-CTP and [^111^In]In-(oxine)_3_. (d) and (e) Viability of cells immediately after labelling, as well as 2, 5 and 20 h after labelling with either (d) [^111^In]In-DTPA-CTP or (e) [^111^In]In-(oxine)_3_. Red line indicates average cell viability at the start of the experiment (before radiolabelling). Statistical significances were determined using a Student's *t* test. For (a), there is substantial statistical significance (*p* < 10^−7^) between [^111^In]In-DTPA-CTP uptake and [^111^In]In^3+^ uptake at each time point, and error bars correspond to a standard deviation of 6 replicates. For (b)–(e), an asterisk denotes a statistical significance (*p < 0.05, **p < 0.01, ***p < 0.001) between [^111^In]In-DTPA-CTP and [^111^In]In-(oxine)_3_ labelling (b) and (c) or between different time points compared to *t* = 0 h (d) and (e), and error bars correspond to standard deviation of 3 biological replicates.

In preliminary studies, 2 × 10^6^ 5T33 cells were incubated with [^111^In]In-DTPA-CTP or [^111^In]In^3+^ (to quantify any uptake of indium-111 that is not CTP-mediated) in RPMI media supplemented with foetal bovine serum, for up to 6 h to establish an optimal cell radiolabelling protocol (Fig. 2(a)). Cell uptake of [^111^In]In^3+^ was negligible. Cell radiolabelling yields for [^111^In]In-DTPA-CTP increased over 0–3 h, to 3.9 ± 0.1%AR (percentage added radioactivity) with no significant gains in yield after 3 h. Thus, an incubation period of 3 h was chosen and this growth medium was used for all subsequent [^111^In]In-DTPA-CTP 5T33 cell radiolabelling experiments.

The indium-111 uptake and efflux, and viability of [^111^In]In-DTPA-CTP-labelled cells were compared with those of cells radiolabelled with [^111^In]In-(oxine)_3_ according to the clinical radiopharmaceutical protocol^19^ (Fig. 2(b)–(e)). In these experiments, 2 MBq of either [^111^In]In-DTPA-CTP or [^111^In]In-(oxine)_3_ were incubated with 1 × 10^5^ 5T33 cells. Cell pellets were obtained by centrifugation, washed and gamma-counted. The cell radiolabelling yield for [^111^In]In-DTPA-CTP was 9.2 ± 3.6%AR – lower than that for [^111^In]In-(oxine)_3_, which was 20.6 ± 6.1%AR (Fig. 2(b)). This corresponded to an average of 1.8 ± 0.6 Bq per cell for [^111^In]In-DTPA-CTP and 4.1 ± 1.0 Bq per cell for [^111^In]In-(oxine)_3_. Both amounts are sufficient for clinical γ-scintigraphy or SPECT imaging.^19^

To measure efflux, cells were washed after radiolabelling and re-incubated in fresh growth medium for 2–20 h. At pre-selected time points, cell pellets were obtained, washed and gamma-counted to quantify the amount of cell-bound [^111^In]In-DTPA-CTP (Fig. 2(c)). Both cell radiolabelling methods resulted in efflux of indium-111 from cells, however retention was significantly higher for cells labelled with [^111^In]In-(oxine)_3_, with 76.0 ± 5.1% of indium-111 remaining associated with cells 2 h after re-incubation and 46.0 ± 9.6% at 20 h. For cells labelled with [^111^In]In-DTPA-CTP, these values were 23.0 ± 5.1% at 2 h and 9.7 ± 0.5% at 20 h.

Cell viability was assessed using a trypan blue exclusion assay (Fig. 2(d) and (e)). Viability for [^111^In]In-DTPA-CTP-labelled cells remained constant throughout the course of the 20 h experiment. Prior to radiolabelling, cell viability measured 89 ± 4%, with 88 ± 2% of cells viable 20 h after radiolabelling. In contrast, viability for [^111^In]In-(oxine)-labelled cells steadily decreased over the course of the experiment. Prior to radiolabelling, cell viability measured 96 ± 0.3%, with 74 ± 6% of cells viable 20 h after radiolabelling.

### SPECT imaging with [^111^In]In-DTPA-CTP

**Fig. 3:**
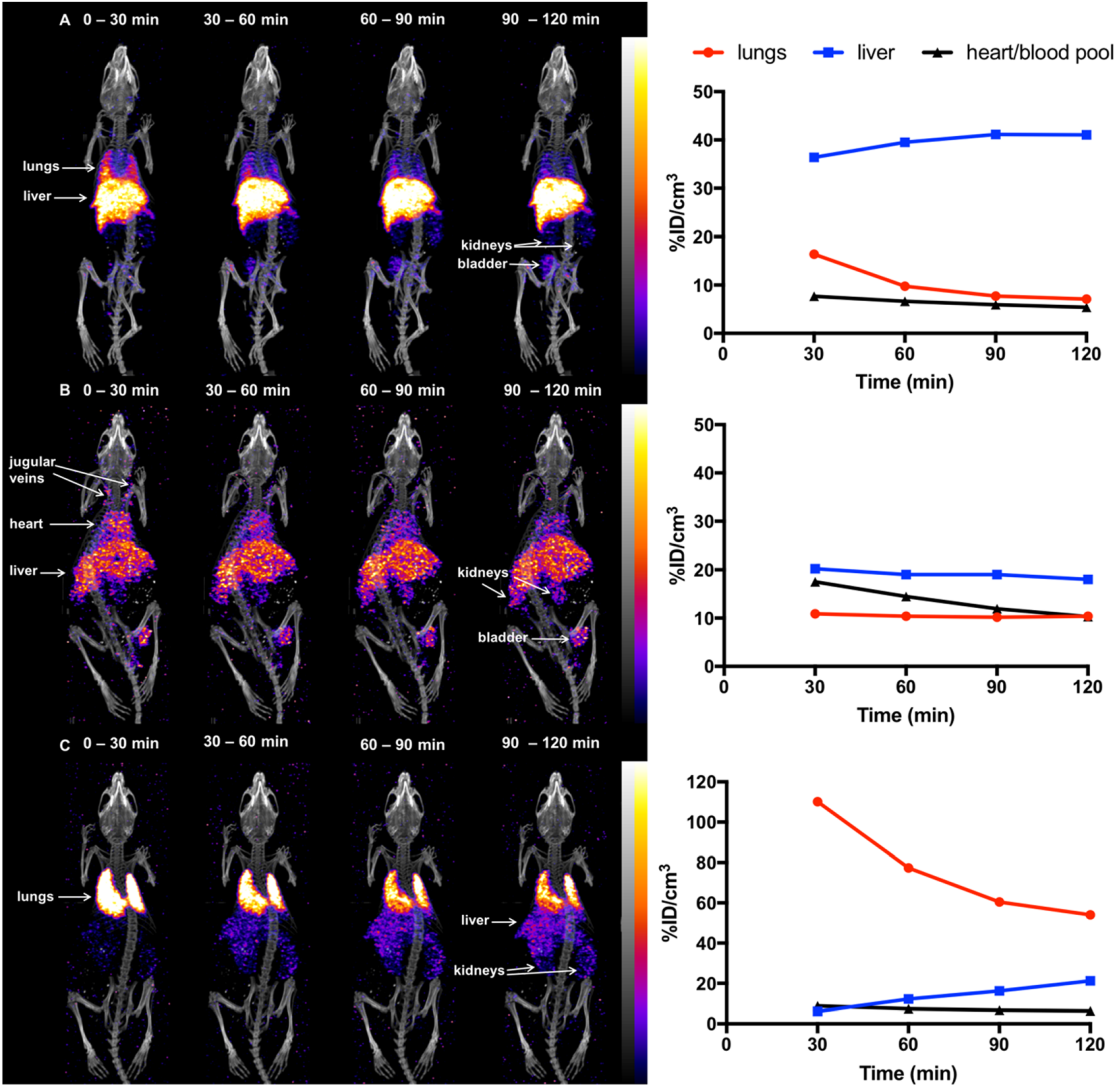
SPECT/CT maximum intensity projections and ^111^In concentrations in specific organs for mice administered (a) [^111^In]In-DTPA-CTP-labelled 5T33 cells (SPECT scale of 0.8–8%ID g^−1^), (b) [^111^In]In-DTPA-CTP (cell-free) (SPECT scale of 1.6–8%ID g^−1^) and (c) [^111^In]In-(oxine)_3_-labelled 5T33 cells (SPECT scale of 1.6–16%ID g^−1^), acquired 0–30 min, 30–60 min, 60–90 min and 90–120 min post-injection.

Previous studies^18,45^ have demonstrated that in mice, 5T33 murine myeloma cells localise in the lungs 0–2 h post-injection (PI), followed by migration to the liver, spleen and bone marrow within 24 h. To probe the ability of [^111^In]In-DTPA-CTP to image the distribution of administered cells *in vivo*, 5T33 cells labelled with [^111^In]In-DTPA-CTP (4 × 10^6^ cells, 7–10 MBq indium-111) were administered intravenously *via* the tail vein to immunodeficient NSG male mice. A SPECT/CT scan was acquired for 2 h immediately after injection (Fig. 3(a)). For comparative purposes, (i) cell-free [^111^In]In-DTPA-CTP (2.5 μg peptide, 3 – 3.3 MBq indium-111) (Fig. 3(b)) and (ii) 5T33 cells labelled with [^111^In]In-(oxine)_3_ (4 × 10^6^ cells, 1.8–2.4 MBq indium-111) (Fig. 3(c)) were administered to mice in parallel, with a 2 h SPECT/CT scan acquired immediately post-injection.

[^111^In]In-(oxine)_3_-labelled 5T33 cells localised to the lungs and then liver 0–2 h PI, consistent with prior studies (Fig. 3(c)). Mice injected with cell-free [^111^In]In-DTPA-CTP exhibited indium-111 activity in the blood pool and liver 0–2 h PI (Fig. 3(b)). Mice injected with [^111^In]In-DTPA-CTP-labelled cells exhibited indium-111 activity in the lungs, liver and blood pool (Fig. 3(a)). For the latter group of mice, SPECT/CT data indicated that although some indium-111 radioactivity accumulated in the lungs 0–30 min PI, this decreased over the course of the next 90 min. In contrast to mice administered [^111^In]In-(oxine)_3_-labelled 5T33 cells, there was significantly greater indium-111 localisation in the liver relative to the lungs.

**Fig. 4:**
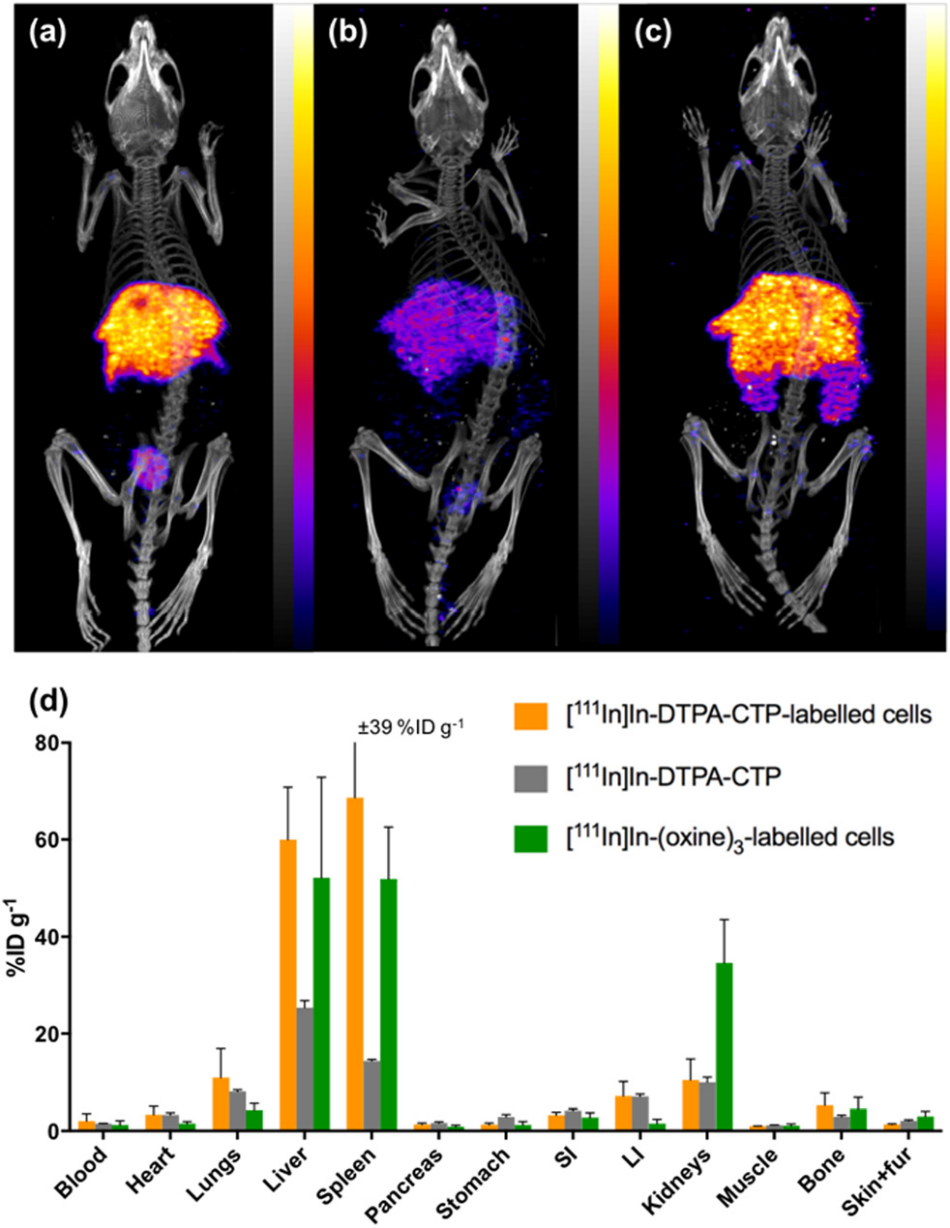
SPECT/CT maximum intensity projections of mice, acquired 1 day post-injection of (a) [^111^In]In-DTPA-CTP-labelled 5T33 cells; (b) [^111^In]In-DTPA-CTP (cell-free); (c) [^111^In]In-(oxine)_3_-labelled 5T33 cells (scale of 0.6–6%ID g^−1^ for all images). In all images, indium-111 is largely concentrated in the liver and spleen. (d) *Ex vivo* biodistribution of ^111^In radioactivity in animals administered [^111^In]In-radiotracers. Error bars represent standard deviation. For animals administered [^111^In]In-DTPA-CTP-labelled 5T33 cells and [^111^In]In-(oxine)_3_-labelled 5T33 cells, *n* = 3, for animals administered cell-free [^111^In]In-DTPA-CTP, *n* = 2.

At 1 day PI, SPECT/CT scans were acquired and all animals were culled, with organs harvested for *ex vivo* radioactivity counting (Fig. 4). There were high concentrations of indium-111 in the liver and spleen of mice administered [^111^In]In-(oxine)_3_-labelled 5T33 cells or [^111^In]In-DTPA-CTP-labelled cells, with no statistically significant differences in these organs across these two groups.

We suggest that some of the initial lung indium-111 uptake observed for animals administered [^111^In]In-DTPA-CTP-labelled cells is a result of accumulation of [^111^In]In-DTPA-CTP-labelled 5T33 cells to the lungs. However, over the course of 0–2 h PI, [^111^In]In-DTPA-CTP dissociates from 5T33 cells, resulting in indium-111 activity in the blood pool and liver 0–2 h PI, similar to the case of mice administered cell-free [^111^In]In-DTPA-CTP. In contrast, [^111^In]In-(oxine)_3_-labelled cells migrate from the lungs to the liver and spleen over a longer time frame, with lower blood pool and liver localisation over 0–2 h PI (Fig. 3) and high liver and spleen localisation by 1 day PI (Fig. 4). This is consistent with data from prior studies.^18,45^ Although animals administered [^111^In]In-DTPA-CTP-labelled 5T33 cells demonstrate high indium-111 radioactivity accumulation in the liver and spleen 1 day PI, this is likely to be a result of accumulation of [^111^In]In-DTPA-CTP that has dissociated from 5T33 cells.

### Protein binding

**Fig. 5:**
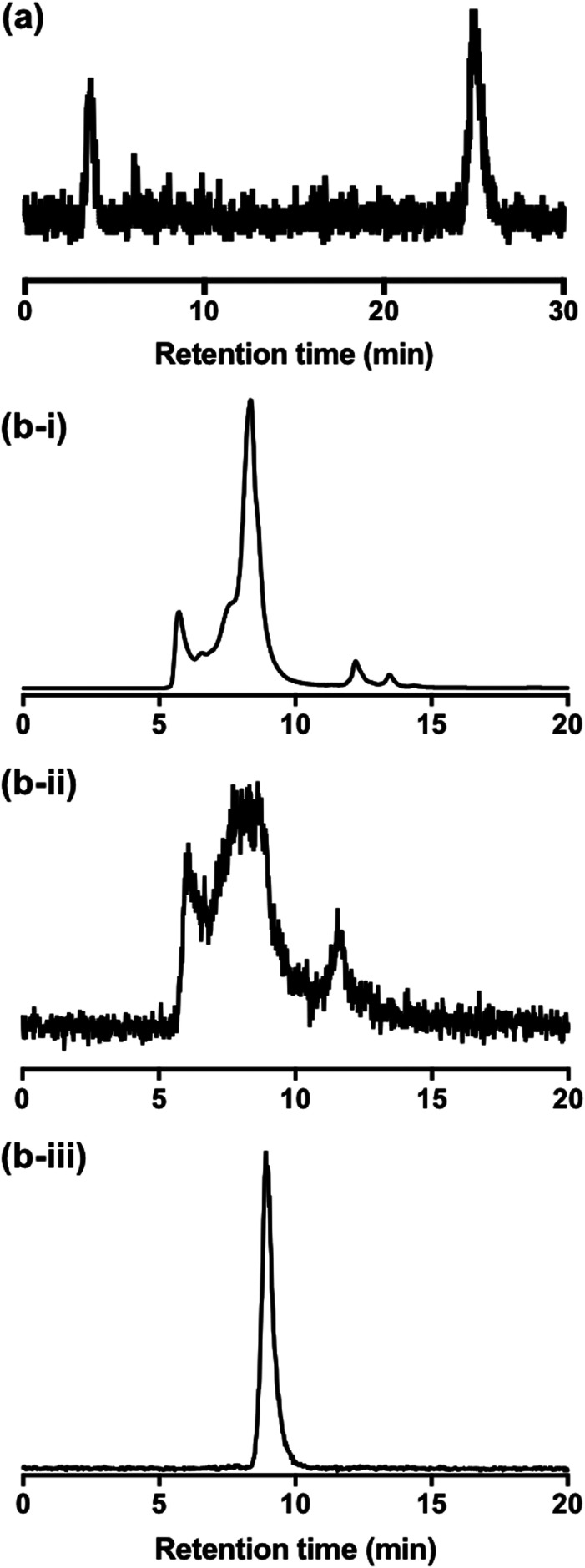
(a) Reverse phase C_8_ HPLC radiochromatogram of [^111^In]In-DTPA-CTP after incubation in murine serum for 24 h, demonstrating that >70% of [^111^In]In-DTPA-CTP remained intact with respect to dissociation of [^111^In]In^3+^ from the DTPA chelator. The signal at 4.1 min corresponds to [^111^In]In^3+^ released from DTPA-CTP. (b)–(i) Size exclusion HPLC UV chromatogram of murine serum (280 nm). (b)–(ii) Size exclusion HPLC radio-chromatogram of a solution of [^111^In]In-DTPA-CTP incubated with murine serum for 24 h. Comparison with the UV chromatogram indicates that [^111^In]In-DTPA-CTP interacts globally with many serum components. (b)-(iii) Size exclusion HPLC radio-chromatogram of a solution of [^111^In]In^3+^ incubated with murine serum for 24 h, indicating that [^111^In]In^3+^ likely binds specifically to a single serum component.

To better understand the behaviour of [^111^In]In-DTPA-CTP in biological media, a solution of [^111^In]In-DTPA-CTP was incubated in murine serum and analysed by both size exclusion HPLC and C_8_ reverse phase HPLC. C_8_ chromatograms demonstrated that over the course of 24 h, greater than 70% of [^111^In]In-DTPA-CTP remained intact (Fig. 5(a)), with respect to the stability of the [^111^In]In-DTPA complex. However, size exclusion chromatograms indicated that [^111^In]In-DTPA-CTP interacted non-specifically with many serum proteins (Fig. 5(b)-i,ii). In contrast, size exclusion chromatograms of solutions of [^111^In]In^3+^ in serum indicated that [^111^In]In^3+^ bound to one specific serum component (Fig. 5(b)-iii).

## Discussion and concluding remarks

Preparation and purification of DTPA-CTP *via* a maleimide-thiol linkage, and subsequent quantitative, room temperature indium-111-radiolabelling of DTPA-CTP, is simple and reproducible, consistent with existing reports on [^111^In]In-DTPA conjugation and radiolabelling methodology.^40–44^ Serum incubation studies are consistent with the known behaviour of [^111^In]In-DTPA bioconjugates.^40,46^ The majority of [^111^In]In-DTPA-CTP is stable in serum with respect to [^111^In]In-DTPA complex stability, although some [^111^In]In^3+^ (less than 30%) is released from DTPA to serum proteins over 24 h. However, [^111^In]In-DTPA-CTP adheres non-specifically to the majority of serum proteins, presumably *via* non-covalent electrostatic and hydrophobic interactions.

The observed serum protein binding of [^111^In]In-DTPA-CTP is consistent with *in vitro* and *in vivo* data: it is likely that serum proteins compete with 5T33 cells for binding [^111^In]In-DTPA-CTP, both in media *in vitro* and in circulation *in vivo*, resulting in the observed dissociation of [^111^In]In-DTPA-CTP from 5T33 cell membranes. It is also likely that *in vivo*, endogenous cells compete with 5T33 myeloma cells for [^111^In]In-DTPA-CTP binding. Thus, *in vivo* the association between [^111^In]In-DTPA-CTP and 5T33 cell membranes is sufficient to allow visualisation of some cells’ localisation to lungs at early time points, but inadequate for quantitative cell tracking over the course of several hours.

*In vitro* results indicate that although [^111^In]In-DTPA-CTP is an inferior cell labelling reagent compared to [^111^In]In-(oxine)_3_ in terms of retention of radiolabel, it can deliver sufficient indium-111 to cells to enable imaging; and that over short periods of time, significant amounts of [^111^In]In-DTPA-CTP are retained by labelled cells. Additionally, [^111^In]In-DTPA-CTP-labelled cells are more viable than cells labelled with [^111^In]In-(oxine)_3_. The lower viability of [^111^In]In-(oxine)_3_-labelled cells could be a result of the relative higher retention of indium-111.

It is also possible that the intracellular location of ^111^In in [^111^In]In-(oxine)_3_-labelled cells,^47^ rather than the cell surface location for [^111^In]In-DTPA-CTP-labelled cells, leads to a corresponding higher degree of radiation damage to nuclear DNA from emitted short-range Auger electrons.^38,39^ The majority of PET or SPECT cell tracking agents rely on cellular internalisation of the radionuclide. There are two examples in which the radiolabelled motifs are covalently appended to endogenous cell surface molecules: (i) a “desferrioxamine” (DFO) chelator that complexes [^89^Zr]Zr^4+^ has been covalently attached *via* a reactive isothiocyanate to cell surface primary amines (for PET cell tracking);^14^ (ii) a fluorescent motif labelled with ^124^I has also been covalently attached to chemically reduced thiol groups on cell surfaces (also for PET cell tracking).^15^ In both instances, radiolabelling of cells did not decrease cell viability, similar to the case of [^111^In]In-DTPA-CTP-labelled cells. However, it is notable that neither ^89^Zr nor ^124^I emit Auger electrons.

Although [^111^In]In-DTPA-CTP-labelled cells show prolonged cell viability, the relatively rapid dissociation of [^111^In]In-DTPA-CTP from cell membranes precludes the use of this agent for quantitative radionuclide cell tracking applications. A CTP–protein bioconjugate, APT070, that prevents organ transplant rejection, is entering clinical trials.^32^ Perfusion of APT070 “paints” endothelial and epithelial cells of transplant organs with a cell-surface protein that inhibits *in vivo* complement activation pathways that lead to transplant rejection. APT070 (with a molecular weight of ∼24 kDa^34^) is significantly larger than In-DTPA-CTP (2886 Da). It is likely that the increased size of the APT070 cell-surface cargo protects the CTP tail from membrane dissociation for sufficient periods of time post-transplantation, enabling amelioration of transplant rejection.

This first cell tracking study on a radiolabelled cell membrane binding peptide has shown that [^111^In]In-DTPA-CTP-labelled myeloma cells can be qualitatively tracked to the lungs using SPECT imaging immediately after intravenous administration. Although [^111^In]In-DTPA-CTP dissociates from cell membranes within 1–2 h post-administration of cells and thus falls short of cell adhesion stability requirements for quantitative long-term cell tracking with SPECT, this proof-of-principle study demonstrates the simplicity and feasibility of using synthetic cell membrane binding/penetrating peptides for radiolabelling cells. Numerous advances in peptide research have enabled efficient cellular attachment and delivery of biomolecules, fluorescent motifs and nanoparticles either *via* cell membrane localisation or cell membrane penetration.^48–51^ Such peptides will be very efficient vectors for stable delivery of radioactive cargo to cells for quantitative whole body cell tracking with SPECT or PET imaging.

## Experimental methods

### CTP-DTPA synthesis

CTP (K(α,ε-bis-myristoyl)-SSKSPSKKDDKKPGD-C(*S*-(2-pyridyldithio))-OH) (prepared by solid phase peptide synthesis as previously described^31^) (1 mg) was reacted with tris(2-carboxyethyl)phosphine (2.5 mM) dissolved in dimethyl sulfoxide/water (15%/85%, 1 mL) for 30 min. The solution was applied to a Waters Sep-Pak C18 Plus Short cartridge and the cartridge was washed with a solution (2.5 mL) of acetonitrile/water (20%/80%) containing trifluoroacetic acid (0.1%). Reduced CTP was eluted from the cartridge with acetonitrile containing trifluoroacetic acid (0.1%), diluted in water (to a volume of 5 mL) and lyophilised. ESI-MS: *m*/*z* for [C_103_H_183_N_23_O_30_S + 3H]^3+^ calc 752.78, found 752.78 (100% signal).

Reduced CTP was dissolved in aqueous ammonium acetate (20 mM) containing dimethylsulfoxide (10%) (1 mL total volume) and added to a solution containing an excess of DTPA-maleimide (50 μL, 20 g mL^−1^) (CheMatech, France). After reaction at room temperature for 30 min, the conjugated peptide was applied to a Sep-Pak C18 Plus Short cartridge (Waters) and the cartridge was washed with a solution (2.5 mL) of acetonitrile/water (35%/65%) containing trifluoroacetic acid (0.1%). Reduced CTP was eluted from the cartridge with acetonitrile containing trifluoroacetic acid (0.1%), diluted in water (to a volume of 5 mL) and lyophilised. ESI-LRMS: *m*/*z* for [(C_123_H_212_N_28_O_41_S) + 4H]^4+^ calc 693.6, found 693.6; [(C_123_H_212_N_28_O_41_S) + 3H]^3+^ calc 924.5, found 924.8. ESI-HRMS: *m*/*z* for [(C_123_H_212_N_28_O_41_S) + Fe + H]^4+^ calc 706.86, found 706.86. We have previously observed Fe^3+^ binding to chelators in our high resolution mass spectrometric analyses.^52^

DTPA-CTP (300 μg) was dissolved in a solution (1 mL) of ammonium acetate (0.2 mM) containing dimethylsulfoxide (10%) and stored at −20 °C for further use.

### [^111^In]In-CTP-DTPA radiosynthesis

A solution of [^111^In]In^3+^ (Mallinckrodt Medical B.V., Petten, Netherlands) (2–6 MBq in 0.1 M HCl) was added to a solution of DTPA-CTP (0.187–1.5 μg, in a final volume of 80 μL of 0.2 M ammonium acetate containing 5% DMSO) and reacted at room temperature for 30 min. An aliquot of the reaction solution was applied to an analytical reverse phase C_8_ HPLC column to determine radiochemical yield. HPLC chromatograms were acquired using an Agilent 1200 LC system, with an Agilent Eclipse XDB-C8 5 μM, 4.6 × 250 mm column coupled to a LabLogic Flow-Count detector with a sodium iodide probe (B-FC-3200). Gradient conditions: 1 mL min^−1^ flow rate, with 100% A at 0 min, with the concentration of B increasing at a rate of 5% min^−1^.

### [^111^In]In-(oxine)_3_ radiosynthesis

[^111^In]In-(oxine)_3_ was prepared using previously described methods.^18^ An aqueous solution of [^111^In]In^3+^ (50 MBq) diluted in water (500 μL) was added to a solution of 8-hydroxyquinoline in chloroform (50 μL, 10 mg mL^−1^). The biphasic mixture was vigorously mixed using a vortex for 5 min. A further aliquot of chloroform (450 μL) was added and the solution mixed using a vortex for 10 min. The chloroform phase was collected and chloroform was evaporated by heating the solution at 50 °C. The residue, containing [^111^In]In-(oxine)_3_ was dissolved in PBS containing dimethyl sulfoxide (2%).

### Cell culture

5T33 myeloma cells stably expressing GFP, originating from C57Cl/KaLwRij strain,^53^ were cultured in RPMI media supplemented with 10% FBS, 1% l-glutamine and 1% penicilin/streptomycin. Cells were sub-cultured every two to three days at a ratio of 1 : 10.

### [^111^In]In-CTP-DTPA cell uptake studies

5T33 myeloma cells (2 × 10^6^, in 200 μL) were incubated in suspension with [^111^In]In-DTPA-CTP (0.1 MBq, 1.2 μg of DTPA-CTP) at 37 °C in RPMI media. The cells were washed with PBS after 1, 2, 3, 4, 5 and 6 h. Activity in both the supernatant (original supernatant and washes) and cell pellet was measured using a γ-counter.

### Comparative uptake, viability and efflux studies

5T33 myeloma cells (10^5^, in 200 μL) were incubated with [^111^In]In-DTPA-CTP (2 MBq, 1.2 μg of DTPA-CTP) for 3 h or [^111^In]In-(oxine)_3_ (2 MBq) for 30 min at 37 °C. [^111^In]In-DTPA-CTP cell labelling was undertaken in serum-containing growth media. [^111^In]In-(oxine)_3_ cell labelling was undertaken in serum-free growth media. After cell labelling, cells were washed twice with PBS and 300 μL of fresh growth media was added. Uptake efficiency was quantified at this time-point by measuring pellet and supernatant in the γ-counter. At 2, 5 and 20 h after addition of fresh media, cells were washed twice and activity in both the supernatant (original supernatant and washes) and pellet was measured using a γ-counter. Efflux was quantified as the activity of the supernatant divided by the total activity (supernatant + pellet). Cell viability was assessed prior to radiolabelling and at each time point by trypan blue exclusion.^54^

### Serum stability studies

[^111^In]In-DTPA-CTP (2 MBq, 1.2 μg peptide) or [^111^In]In^3+^ (2 MBq) were incubated in 200 μL mouse serum for 24 h and subsequently analysed using both C_8_ and size exclusion HPLC. For C_8_ analysis, serum proteins were precipitated by addition of 200 μL acetonitrile to each sample, followed by centrifugation and separation of supernatant from pellet. The supernatant was applied to an analytical reverse phase C_8_ HPLC column using conditions described above. For size exclusion, the sample was centrifuged to remove any particulate matter, and the supernatant was applied to an analytical size exclusion column. Analytical size exclusion radio-HPLC traces were acquired using an Agilent 1200 Series HPLC system and a Phenomenex Biosep 2000 (300 × 7.8 mm) size exclusion column, coupled to a LabLogic Flow-Count detector with a sodium iodide probe (B-FC-3200). Isocratic mobile phase conditions: 1 mL min^−1^ flow rate of phosphate buffered saline solution.

### SPECT/CT imaging and biodistribution

Animal imaging studies were ethically reviewed by an Animal Welfare & Ethical Review Board at King's College London and carried out in accordance with the Animals (Scientific Procedures) Act 1986 (ASPA) UK Home Office regulations governing animal experimentation. NSG male mice (6 weeks old) were injected intravenously under isoflurane anesthesia, with either [^111^In]In-DTPA-CTP-labelled 5T33 cells (4 × 10^6^ cells, 7–10 MBq, *n* = 3), [^111^In]In-DTPA-CTP (cell-free) (2.5 μg peptide, 3–3.3 MBq, *n* = 2), or [^111^In]In-(oxine)_3_-labelled 5T33 cells (4 × 10^6^ cells, 1.8–2.4 MBq, *n* = 3) *via* the tail vein. Animals were maintained under isoflurane inhalation anaesthesia (2– 2.5% Isoflurane in air) for 2 h post-injection. For one animal in each of these groups, a SPECT/CT scan was acquired on a dedicated small animal SPECT system, NanoSPECT/CT Silver Upgrade (Mediso Ltd, Budapest, Hungary), calibrated for indium-111. The whole body SPECT scan time was 30 min × 4, (conducted sequentially) with a frame time of 40 s (using a 4-head scanner with 4 × 9 [1.4 mm] pinhole collimators in helical scanning mode) followed by a helical CT (45 kVP X-ray source, 1000 ms exposure time in 180 projections over 7.5 min). After this, animals were allowed to recover. SPECT/CT scans were acquired again, 1 day PI, after which all animals were culled (by increasing the dose of anaesthesia followed by cervical dislocation to confirm death) and organs/tissues harvested, weighed and radioactivity counted using a gamma counter. SPECT/CT images were reconstructed in a 256 × 256 matrix using HiSPECT (ScivisGmbH), a reconstruction software package and visualised and quantified using VivoQuant v.3.0 software (InVicro LLC., Boston, USA).

## Abbreviations

%AR: Percentage added radioactivity
%ID: Percentage injected dose
CAR: Chimeric antigen receptor
CT: Computed tomography
CTP: Cytotopic peptide
DTPA: Diethylenetriamine pentaacetate
HPLC: High performance liquid chromatography
PET: Positron emission tomography
PI: Post-injection
SPECT: Single photon emission computed tomography

## Declarations

### Ethical approval and consent to participate

All animal experiments on mice complied with the Animals (Scientific Procedures) Act (UK 1986) and Home Office (UK) guidelines. No human participants were involved in this study.

## Author contributions

J. P. contributed to the study design, prepared the radiotracers, undertook *in vitro* and *in vivo* experiments, analysed data and co-wrote the manuscript, J. E. B, T. T. P., A. V. and K. S. contributed to the study design and experiments, P. C. contributed to the study design and undertook preliminary *in vitro* experiments, G. E. D. M and P. B. contributed to the study design, R. A. G. S. contributed peptide and contributed to the study design, M. T. M. led study design, supervised the project and led compilation of the manuscript.

## Conflicts of interest

There are no conflicts to declare.

## Supplementary Material
